# Diagnostic Significance of Serum Galectin-3 in Hospitalized Patients with COVID-19—A Preliminary Study

**DOI:** 10.3390/biom11081136

**Published:** 2021-08-01

**Authors:** Beata Kuśnierz-Cabala, Barbara Maziarz, Paulina Dumnicka, Marcin Dembiński, Maria Kapusta, Monika Bociąga-Jasik, Marek Winiarski, Aleksander Garlicki, Tomasz Grodzicki, Michał Kukla

**Affiliations:** 1 Department of Diagnostics, Chair of Clinical Biochemistry, Faculty of Medicine, Jagiellonian University Medical College, Krakow 31-066, Poland; beata.kusnierz-cabala@uj.edu.pl (B.K.-C.); barbara.maziarz@uj.edu.pl (B.M.); maria.kapusta@uj.edu.pl (M.K.); 2 Department of Medical Diagnostics, Faculty of Pharmacy, Jagiellonian University Medical College, Krakow 30-688, Poland; 3 2nd Department of General Surgery, Faculty of Medicine, Jagiellonian University Medical College, Krakow 30-688, Poland; m.dembinski@uj.edu.pl (M.D.); marek.winiarski@uj.edu.pl (M.W.); 4 Hepatology and Infectious Diseases, Chair of Gastroenterology, Faculty of Medicine, Jagiellonian University Medical College, Krakow 30-688, Poland; monika.bociaga-jasik@uj.edu.pl (M.B.-J.); aleksander.garlicki@uj.edu.pl (A.G.); 5 Department of Internal Medicine and Geriatrics, Faculty of Medicine, Jagiellonian University Medical College, Kraków 30-688, Poland; tomasz.grodzicki@uj.edu.pl (T.G.); michal.kukla@uj.edu.pl (M.K.); 6 Department of Gastroscopy, University Hospital in Kraków, Krakow 30-688, Poland

**Keywords:** galectin-3, soluble fms-like tyrosine kinase-1, SARS-CoV-2, COVID-19, inflammation

## Abstract

Severe coronavirus disease 2019 (COVID-19) is associated with hyperinflammation leading to organ injury, including respiratory failure. Galectin-3 was implicated in innate immunological response to infections and in chronic fibrosis. The aim of our preliminary study was the assessment of the diagnostic utility of serum galectin-3 in patients with COVID-19. The prospective observational study included adult patients admitted with active COVID-19 and treated in tertiary hospital between June and July 2020. The diagnosis was confirmed by the quantitative detection of nucleic acid of severe acute respiratory syndrome coronavirus 2 in nasopharyngeal swabs. Galectin-3 was measured by enzyme immunoassay in serum samples obtained during the first five days of hospital stay. We included 70 patients aged 25 to 73 years; 90% had at least one comorbidity. During the hospital stay, 32.9% were diagnosed with COVID-19 pneumonia and 12.9% required treatment in the intensive care unit (ICU). Serum galectin-3 was significantly increased in patients who developed pneumonia, particularly those who required ICU admission. Positive correlations were found between galectin-3 and inflammatory markers (interleukin-6, C-reactive protein, ferritin, pentraxin-3), a marker of endothelial injury (soluble fms-like tyrosine kinase-1), and a range of tissue injury markers. Serum galectin-3 enabled the diagnosis of pneumonia with moderate diagnostic accuracy and the need for ICU treatment with high diagnostic accuracy. Our findings strengthen the hypothesis that galectin-3 may be involved in severe COVID-19. Further studies are planned to confirm the preliminary results and to verify possible associations of galectin-3 with long-term consequences of COVID-19, including pulmonary fibrosis.

## 1. Introduction

A spectrum of complications observed in patients with coronavirus disease 2019 (COVID-19) is not restricted to the widely recognized severe acute respiratory syndrome. The complications may include exacerbation of comorbidities, coagulopathies, bacterial superinfections with increased risk of sepsis, and multiple organ failure (MOF) [1]. Therefore, a prognosis and monitoring of patients’ state require a wide panel of laboratory tests. Although some work has been done to reach a consensus regarding the laboratory panel in monitoring COVID-19 patients, there is currently no agreement on which tests should be included. However, considering the pathophysiology of COVID-19 in adults, the list usually includes complete blood count and hematological indices, acute phase reactants, markers of tissue injury including cardiac biomarkers, renal and liver function tests, coagulation profile, and arterial blood gases [2,3,4].

Considering the dynamic changes in clinical state of patients with severe COVID-19, there is a need to optimize the list of laboratory tests balancing the time-relevant and potentially time-limited parameters. An example of such an attempt is the recommendation for minimal laboratory testing panels in patients with COVID-19 published by Favaloro et al. [5]. The recent meta-analysis by Moutchia et al. [4] summarizes the current data on the diagnostic utility of a range of laboratory tests in severe to critical COVID-19 patients. In patients with severe to critical COVID-19, significant prognostic parameters include a decrease in serum albumin in line with an increase in serum concentrations of acute phase proteins (C-reactive protein, CRP, and ferritin), interleukin 6 (IL-6), and procalcitonin (PCT) [2]; an increase in serum activity of lactate dehydrogenase; increased plasma fibrinogen and D-dimer; and hypoxia [2,4,5].

Research continues regarding the laboratory markers related to the severity of COVID-19. Recent studies evaluating the inflammatory markers indicate the prognostic value of long pentraxin, known as pentraxin-3 (PTX-3), produced by endothelial cells, monocytes, macrophages, fibroblasts, and smooth muscle cells as a part of innate immunological response to infections [6,7]. As PTX-3 production is driven by other factors than IL-6, it may be a marker complementary to IL-6 or CRP [6]. The high incidence of thromboembolic events in COVID-19 patients point to the endothelial injury developing in the course of the disease. This may be caused by both the cytokine storm and the direct action of severe acute respiratory syndrome coronavirus 2 (SARS-CoV-2) entering endothelial cells through the binding of viral spike protein with the cell membrane angiotensin-converting enzyme 2 (ACE-2) [8,9]. Highly increased D-dimer has been consistently associated with adverse prognosis in COVID-19 [10,11]. Angiopoietin-2, a marker of endothelial activation and injury observed in acute inflammatory states, has been associated with the need for intensive care unit (ICU) admission in COVID-19 [12]. In the present study, we included another laboratory marker associated with endothelial dysfunction, the soluble fms-type tyrosine kinase 1 (sFlt-1), which we have previously shown to correlate with serum angiopoietin-2 and to predict organ failure and coagulopathy in acute pancreatitis [13,14].

Galectin-3 is a carbohydrate-binding protein (a lectin) associated with acute and chronic inflammation, innate immunity, oxidative stress, and fibrosis [15,16]. Galectin-3 is released by inflammatory cells, mainly macrophages, endothelial cells, and epithelial cells. It is found in spleen, stomach, colon, liver, kidney, heart, uterus, ovary, pancreas, and lungs (alveolar cells) [15,17]. Galectin-3 has been previously studied in relation to several viral infections in human, including human immunodeficiency virus, herpesvirus-1 infection, influenza, early herpes zoster neuralgia, and postherpetic neuralgia [15,17,18,19,20].

The proinflammatory action of galectin-3 is associated with the activation of the transcription factor NF-κB and induction of tumor necrosis factor α (TNF-α) and IL-6, regulation of cell adhesion, promotion of cell activation and chemotaxis, and regulation of cell growth and apoptosis [17,21,22]. In severe COVID-19, the systemic hyperinflammation with the cytokine storm has been implicated as a cause of respiratory failure and other organs’ injury [23,24]. Moreover, there is a structural similarity between human galectin-3 and the N-terminal domain of the spike proteins of β-coronaviruses [15,22,25,26]. It has been hypothesized that the galectin’s inhibitors may also bind the S1-N-terminal domain of β-coronaviridae, which could partially inhibit the affinity of S1 to cell membrane components [22]. Therefore, galectin-3 has been suggested as a significant mediator and a potential drug target in COVID-19 [15,21,22]. However, we are only aware of a single published report on serum concentrations of galectin-3 in relation to COVID-19 severity (PubMed search on 5 May 2021) [27].

The aim of our preliminary study was the assessment of the diagnostic utility of serum galectin-3 concentrations in patients with COVID-19 of various severity, in comparison to other relevant markers, including a wide panel of recommended laboratory tests, and additionally serum concentrations of PTX-3 and sFlt-1.

## 2. Materials and Methods

### 2.1. Study Protocol and Patients

The prospective observational study included adult patients admitted with active COVID-19 and treated in University Hospital in Kraków, Poland, between June and July 2020. The diagnosis was confirmed in each patient by the quantitative detection of nucleic acid of SARS-CoV-2 in nasopharyngeal and oropharyngeal swabs collected during infection. Detection of SARS-CoV-2 RNA was carried out with a real-time RT PCR test on the Cobas 6800/8800 Systems (Roche Diagnostics, Mannheim, Germany) in the Microbiology Department of the University Hospital in Krakow, Poland. For monitoring the entire sample’s preparation and PCR amplification process, the RNA Internal Control was used. The results of laboratory tests described below were compared with the clinical data collected as a part of routine care during the patients’ hospital stay. The clinical data included patients’ age, BMI, the signs and symptoms observed at admission, the comorbidities, the occurrence of pneumonia, the need for treatment in the ICU during the hospital stay, the length of ICU stay, the time from admission to hospital to the transfer to ICU, and the total length of the hospital stay. The data on comorbidities were summarized using the Charlson comorbidity index [28].

The study protocol was approved by the Jagiellonian University Bioethics Committee (approval number KBET 1072.6120.157.2020 issued on 25 June 2020). All the study participants gave written informed consent to participate in the study.

### 2.2. Laboratory Tests

The blood was collected from patients as a part of routine hospital care. The laboratory tests were performed in K_2_EDTA blood (complete blood count), serum (biochemistry and immunochemistry), and citrate-anticoagulated plasma (coagulation tests). The routine laboratory tests included complete blood counts with leukocyte differential (Sysmex XN-2000 hematology analyzer, Sysmex Corporation, Cobe, Japan), biochemistry (serum glucose, bilirubin, urea, creatinine, total protein, albumin, sodium, potassium, iron, lipid profile, alanine aminotransferase, aspartate aminotransferase, lactate dehydrogenase, alkaline phosphatase, creatine kinase, gamma glutamyl-transpeptidase) and immunochemistry (troponin, myoglobin, N-terminal pro-B-type natriuretic peptide, NT-proBNP, ferritin, CRP, IL-6; Cobas PRO 6000 analyzer, Roche Diagnostics, Mannheim, Germany), and coagulation tests (prothrombin time, fibrinogen, D-dimer; BCS XP analyzer, Siemens Healthcare, Erlangen, Germany). All the routine laboratory tests were done in the Diagnostic Department of University Hospital, Krakow, Poland, on the day of blood collection.

Additional tests were performed using the excess of serum that remained after routine testing. For these tests, we used samples collected from patients with confirmed SARS-CoV-2 infection who gave their consent to be included into the study. The samples were collected between day 0 and day 5 after admission to hospital. A single sample was analyzed per patient. The samples were aliquoted and kept frozen at –80 ˚C for maximum of 1 month until the measurements were performed in series. Soluble fms-like tyrosine kinase-1 (sFlt-1) was measured in serum using a Cobas PRO 6000 analyzer (Roche Diagnostics, Mannheim, Germany); the serum concentrations of sFlt-1 in healthy subjects were 63–108 pg/mL. Serum concentrations of pentaxin-3 (PTX-3) and galectin-3 were measured by enzyme immunoassays (ELISA) using commercially available kits (Pentraxin 3 Human ELISA, BioVendor, Brno, Czech Republic; and Quantikine ELISA Human Galectin-3 Immunoassay, R&D Systems, McKinley Place, MN, USA, respectively). The minimum detectable dose of human galectin-3 ranged from 0.003 to 0.085 ng/mL; for PTX-3, the limit of detection was 0.022 ng/mL. The reference values determined by the manufacturers of the kits were 0.721–1.558 ng/mL (men) and 0.733–2.519 (women) for PTX-3 and 2.40–15.7 ng/mL for galectin-3, respectively. Patients’ samples were tested in series, according to the manufacturer’s instructions. The readings were made with an automatic microplate reader (Automatic Micro ELISA Reader ELX 808, BIO-TEK Instruments Inc., Winooski, VT, USA).

Serum sFlt-1, PTX-3, and galectin-3 were also measured in serum samples collected from a total of 31 healthy volunteers in order to obtain the control values: sFlt-1 concentrations were measured in 21 samples, while galectin-3 and PTX-3 concentrations were measured in 10 samples. Adult individuals in the age range (34–71 years) matched with the age range of patients, and 16 women and 15 men were recruited. The healthy volunteers did not show and signs or symptoms of disease at sample collection, and had no history of chronic diseases (including diabetes, gastrointestinal, renal, or systemic diseases or chronic infections).

### 2.3. Statistical Analysis

The statistical analysis was performed with the use of Statistica v13.3 (Tibco Software Inc., Tulsa, OK, USA). The categorical data were summarized as the number of patients (percentage of the specified group). The contingency tables were analyzed using Pearson chi-squared test. Quantitative data with normal or non-normal distribution were presented as mean and standard deviation (SD) or median and interquartile range (IQR), respectively. The selected subgroups were compared with t-test or Mann–Whitney test according to distribution of the dependent variable. The Kruskal–Wallis test was used to compare serum galecin-3 concentrations (non-normally distributed variable) between multiple categories. The Spearman rank correlation coefficient was used to assess correlations between serum galectin-3 and the studied variables. The receiver operating characteristics (ROC) curves were analyzed to assess the diagnostic accuracy of studied tests. The area under the ROC curve (AUC) with a 95% confidence interval served as the measure enabling the comparison of studied tests. The diagnostic cut-off values for serum galectin-3 were calculated by maximizing the Youden index. Multiple logistic regression was used to verify the association between galectin-3 and the severity of COVID-19 after adjustment for predefined covariates (age and comorbidities); the alternative model described in the text of results included diabetes, which was significantly associated with the outcome in simple analysis. The statistical tests were two-tailed, and the results were considered significant at a *p*-value below 0.05.

## 3. Results

### 3.1. Characteristics of Patients

We included 70 patients (43 women and 27 men) admitted to hospital with confirmed COVID-19 (Table 1). The age of the patients ranged from 25 to 73 years; 34 patients (48.6%) were above 60 years of age. Among 40 patients with available data on body mass index (BMI), 19 (47.5%) were overweight and 13 (32.5%) were obese. The majority of patients (n = 63, 90%) had at least one preexisting comorbidity, most commonly hypertension, diabetes, and thyroid diseases (Table 1). There was one patient with type 1 diabetes, 13 had type 2 diabetes, and 2 had post-steroid diabetes mellitus.

On admission, hemoglobin oxygen saturation was < 95% in 17 (24.3%) patients, while 29 (41.4%) displayed dyspnea. Cough and weakness were the most commonly reported initial symptoms (Table 1). The average results of most laboratory tests performed on the first days of hospital stay fell within the reference ranges (Table 2). The serum concentrations of sFlt-1 was significantly higher in COVID-19 patients as compared to healthy individuals (Table 2); however, we did not observe significant differences in serum galectin-3 and PTX-3. No significant differences were observed in sex and age between patients and healthy controls.

### 3.2. Association of Galectin-3 with the Severity of COVID-19

**Figure 1:**
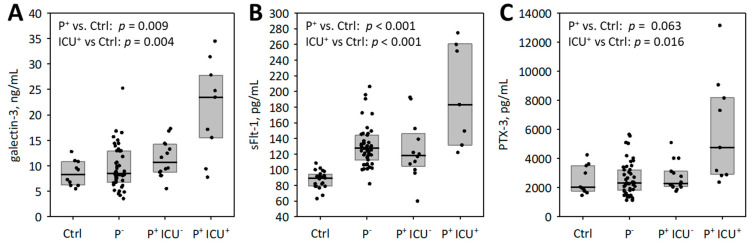
Serum concentrations of galectin-3 (**A**), soluble fms-like tyrosine kinase-1 (sFlt-1; **B**), and pentraxin-3 (PTX-3; **C**) among studied patients with COVID-19 of various severity in comparison to healthy controls (Ctrl). P^−^ denotes COVID-19 patients without pneumonia; P^+^ indicates those with pneumonia, including patients not requiring (ICU^−^) and those requiring treatment in the intensive care unit (ICU^+^). Data are shown as median (line), interquartile range (box), and raw data (points); *p*-values from the Mann–Whitney test are reported.

The length of hospital stay in the studied group ranged from 4 to 111 days (median: 27; Q1: 16; Q3: 39 days). During the hospital stay, 23 (32.9%) patients were diagnosed with COVID-19 pneumonia confirmed by computed tomography imaging. Among the patients with pneumonia, nine (39.1% of those with pneumonia; 12.9% of the whole studied group) required treatment in the ICU. The diagnosis of pneumonia was confirmed during the first five days of hospital stay in all patients. In the nine patients who required intensive care, the time from admission to the transfer to ICU ranged between 1 and 29 days (median 2 days), and the time of stay in ICU ranged between 4 and 55 days (median 18 days). The patients who developed pneumonia were characterized by higher baseline body temperature and lower hemoglobin oxygen saturation (Table 3). Moreover, the patients with pneumonia had higher platelet counts; higher serum activities of gamma-glutamyl transpeptidase (GGT) and lactate dehydrogenase (LDH); longer prothrombin times; higher serum concentrations of IL-6, ferritin, PTX-3, and galectin-3; and lower serum albumin, total protein, and high-density lipoprotein (HDL) cholesterol (Table 3). Although serum galectin-3 in the whole studied group of patients with COVID-19 did not differ as compared to healthy subjects (Table 2), a significant difference was observed between the patients with pneumonia and the healthy persons (*p* = 0.009; Figure 1). No significant difference in serum sFlt-1 was observed between COVID-19 patients who developed pneumonia and those who did not (median 132 and 114 pg/mL, respectively; *p* = 0.4).

The patients who required ICU treatment during the hospital stay were characterized by lower diastolic blood pressure and hemoglobin oxygen saturation, as well as a range of laboratory abnormalities, including anemia; increased white blood cell (WBC), neutrophil, and monocyte counts; increased platelet counts; increased serum concentrations of CRP, IL-6, ferritin, D-dimer, PTX-3, sFlt-1, and galectin-3; high serum activities of GGT and LDH; and low concentrations of albumin and HDL-cholesterol (Table 4; Figure 1). Among comorbidities, diabetes was significantly associated with the need for intensive care (diabetes was diagnosed in 66.7% of patients treated in the ICU and 18.0% of patients who did not require intensive care; *p* = 0.002).

Both serum galectin-3 and PTX-3 (but not sFlt-1) concentrations positively correlated with the length of hospital stay (R = 0.28; *p* = 0.023 and R = 0.29; *p* = 0.017, respectively). Moreover, in patients who required intensive care, PTX-3 concentrations significantly correlated with time in ICU (R = 0.70; *p* = 0.035).

### 3.3. Correlations of Galectin-3 with Clinical Characteristics of Patients and Laboratory Test Results

**Figure 2:**
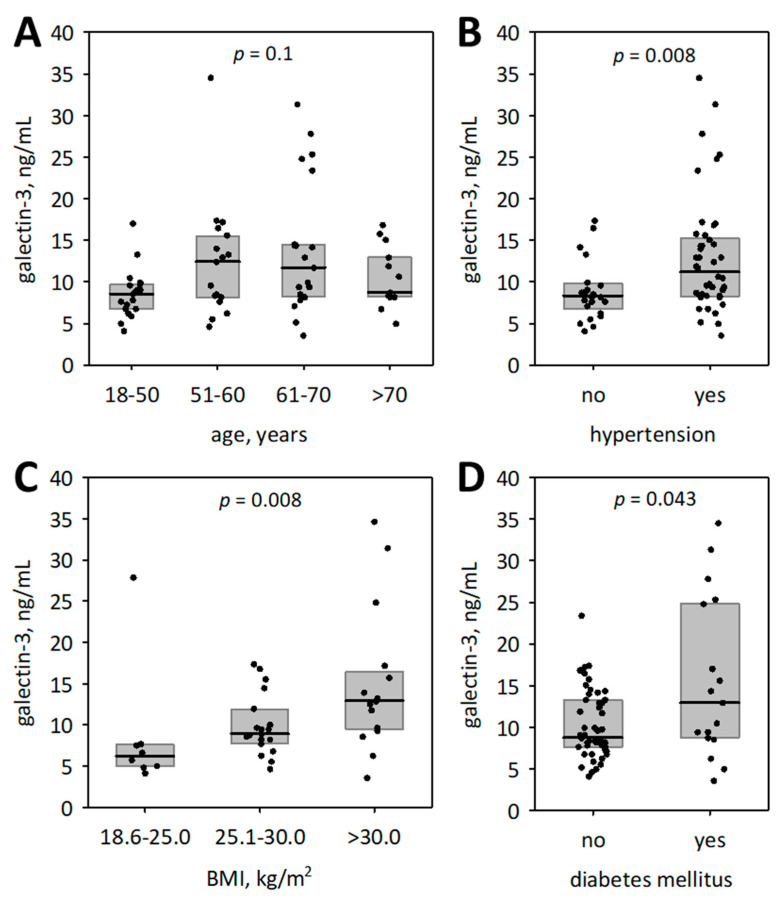
Serum galectin-3 concentrations according to age categories (**A**), the status of hypertension (**B**), overweight/ besity (**C**), and diabetes mellitus (**D**). Medians are represented by the central lines; interquartile ranges are represented by the boxes; raw data are shown as points; *p*-values were calculated using Kruskal–Wallis ANOVA or Mann–Whitney test.

We did not observe significant association between serum galectin-3 and sex (*p* = 0.6) or age of the studied patients with COVID-19 (R = 0.18; *p* = 0.1). Higher serum galectin-3 was observed among patients with hypertension and diabetes, as well as among overweight or obese subjects (Figure 2). We did not observe significant difference in serum galectin-3 concentrations between patients with or without liver abnormalities (defined as ALT, GGT, or total bilirubin above the upper reference limit) (median concentrations 9.90 and 8.86 ng/mL, respectively; *p* = 0.064). We observed significant correlations between galectin-3 and hemoglobin oxygen saturation and diastolic blood pressure on admission (Table 5). Higher Charlson comorbidity index, higher BMI, lower HDL-cholesterol, and higher triglycerides were associated with higher galectin-3 (Table 5). Galectin-3 was positively correlated with most studied inflammatory markers (CRP, IL-6, PTX3, WBC, ferritin), and negatively correlated with serum albumin (Table 5); however, it did not correlate with neutrophil, lymphocyte, monocyte count, or neutrophil/lymphocyte ratio (NLR). Patients with lower RBC, hemoglobin, and hematocrit had higher galectin-3 (Table 5). We observed no correlations between galectin-3 and platelet count, prothrombin time, and D-dimer concentrations. However, serum galectin-3 was strongly positively correlated with sFlt-1 (Table 5). In addition, sFlt-1 was positively correlated with PTX-3 (R = 0.49; *p* < 0.001). Although galectin-3 correlated significantly with serum urea, the correlation with serum creatinine was non-significant (R = 0.13; *p* = 0.3). Weak negative correlation was observed with serum sodium. There was strong positive correlation with LDH, and weak correlations with aspartate aminotransferase and GGT (Table 5).

### 3.4. Diagnostic Usefulness of Galectin-3 in Moderate to Severe COVID-19

**Figure 3:**
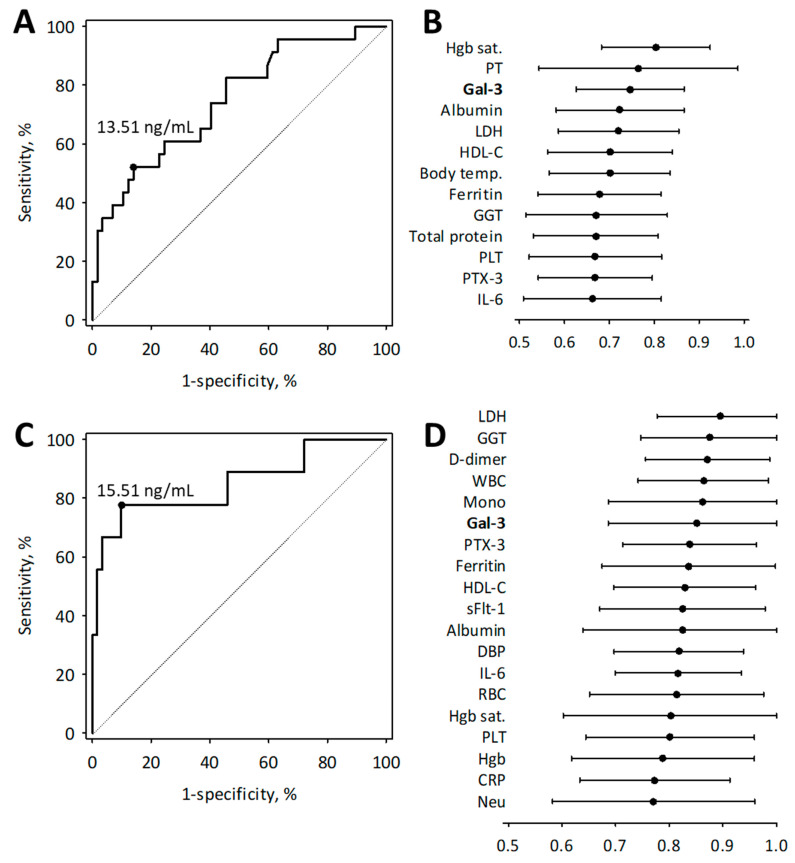
The ROC curves showing the diagnostic accuracy of serum galectin-3 concentrations for the diagnosis of COVID-19 pneumonia (**A**) and the need for of ICU admission (**C**) together with the cut-off values selected using the maximum Youden index. The comparison of area under the ROC curve values (AUC) of the tests with the diagnostic usefulness in the diagnosis of COVID-19 pneumonia (**B**) and the need for ICU admission (**D**): the estimated AUC value (central points) are shown with the 95% confidence intervals (whiskers). Abbreviations: see Table 1 and Table 2; Hgb sat., hemoglobin oxygen saturation; PT, prothrombin time; Gal-3, galectin-3; LDH, lactate dehydrogenase; HDL-C, HDL-cholesterol; temp., temperature; GGT, gamma-glutamyl transferase; PLT, platelet count; PTX-3, pentraxin-3; IL-6, interleukin-6; Mono, monocyte count; DBP, diastolic blood pressure; Hgb, hemoglobin; CRP, C-reactive protein; Neu, neutrophil count.

The analysis of the ROC curves confirmed that serum galectin-3 measured in hospitalized patients with COVID-19 had moderate diagnostic accuracy for COVID-19 pneumonia and high diagnostic accuracy for the need for ICU treatment (Figure 3). However, we did not find statistically significant differences between the AUC values calculated for the studied diagnostic markers (presented in Figure 3). Serum galectin-3 above 13.51 ng/mL was associated with COVID-19 pneumonia with a diagnostic sensitivity of 52% and specificity of 86%, while concentrations above 15.51 ng/mL were associated with the need for ICU admission with a diagnostic sensitivity of 78% and specificity of 90% (Figure 2). In multiple logistic regression, galectin-3 was significantly associated with pneumonia and ICU treatment independently of age and Charlson comorbidity index (Table 6). In addition, galectin-3 was associated with the need for ICU treatment independently of diabetes (odds ratio: 1.26; 95% confidence interval: 1.07–1.48; *p* = 0.004).

In the ROC analysis, serum PTX-3 proved a significant diagnostic marker of COVID-19 pneumonia and the need for ICU admission, while serum sFlt-1 showed some value in the diagnosis of the need for ICU treatment (Figure 2).

## 4. Discussion

To the best of our knowledge, this is the second report showing the positive association between serum galectin-3 concentrations and more severe courses of COVID-19. We found significantly higher serum galectin-3 in patients with COVID-19 pneumonia and in those requiring treatment in the ICU as compared to the cases with less severe disease and to the healthy population. Highly significant positive correlations were shown between serum galectin-3 concentrations and the studied inflammatory markers, including IL-6, PTX-3, and ferritin, and the endothelial dysfunction marker, sFlt-1. A negative correlation was observed between galectin-3 and albumin, the negative acute phase protein. We have shown that the diagnostic accuracy of serum galectin-3 for severe COVID-19 (pneumonia and the need for ICU admission) is high and comparable with other relevant diagnostic markers.

In October 2020, De Biasi et al. [29] reported a detailed analysis of immune alterations in lymphopenic patients with COVID-19 pneumonia recruited in Italy between March and May 2020. The report included the observation of significantly higher plasma galectin-3 concentrations in 23 COVID-19 patients as compared to 15 healthy controls. Our study confirmed the increased serum galectin-3 in patients with COVID-19 pneumonia, especially those requiring intensive care. However, in our study, serum galectin-3 did not differ significantly between the whole group of patients and the controls. This discrepancy between our results and those of De Biasi et al. [29] may be most likely explained by the difference in disease severity between our patients and those recruited by De Biasi et al. (all of whom were diagnosed with COVID-19 pneumonia). Additionally, the two studies used different laboratory methods to measure galectin-3 concentrations (De Biasi et al. measured plasma concentrations with a Luminex assay, while we used a serum and enzyme-linked immunosorbent assay). In the study of De Biasi et al. [29], patients were not stratified according to COVID-19 severity.

The report of Kazancioglu et al. [27] was published online on 31 March 2021. The authors measured serum galectin-1, galectin-3, and prostaglandin E in the samples of 84 patients with confirmed COVID-19, treated in a tertiary hospital in Turkey, and 58 healthy controls. Treatment in ICU was necessary in 22% of their patients. Galectin-3 was measured with ELISA; however, using a different kit (i.e., Bioassay Technology Laboratory, Shanghai, China) than in our study. The patients with severe disease (defined based on respiratory function: breath rate > 30/min, hemoglobin oxygen saturation < 94%, or arterial to inspired oxygen ratio < 300, or pulmonary infiltrates > 50%) had higher serum galectin-3 than those with milder disease, and both groups showed higher concentrations compared to controls (median concentrations: 415, 326, and 243 pg/mL, respectively). The authors did not observe significant correlations between serum galectin-3 and inflammatory markers (CRP, IL-6, ferritin, procalcitonin), anemia, coagulation abnormalities, or serum enzymes (alanine or aspartate transaminases, LDH, creatinine kinase), even though the results of these tests differed significantly between severe and non-severe patients [27]. The authors did not report data on kidney function. Moreover, Kazancioglu et al. [27] did not assess the diagnostic accuracy of serum galectin-3, and did not compare it with other laboratory tests. Our study was consistent with Kazancioglu et al. [27] in the main finding; i.e., both showed significantly increased serum galectin-3 in more severe COVID-19. However, the significant correlations of galectin-3 with other markers of severity were only detected in our study. This may be associated with different timeframes of blood collection with respect to the beginning of the disease, or with different patients’ characteristics, both depending on the specificity of local organization of hospital care of patients with COVID-19. Alternatively, there may be significant differences in the performance characteristics of ELISA kits used to measure galectin-3.

Galectin-3 is involved in innate immunological reactions to infections, serving as a pattern-recognition receptor, a danger-associated molecular pattern molecule, and an immunomodulator [30]. It has been shown to induce cytokine release in human cells, including IL-1β, IL-6, and tumor necrosis factor-α [22,31,32]. There are numerous reports linking galectin-3 with the course of various viral infections. The genetic polymorphisms of the galectin-3-encoding gene affected the course of enterovirus 71 infection in children, which was partially associated with diverse viral replication according to the gene variant [33]. Increased serum galectin-3 has been observed in patients with varicella-zoster virus infection and patients with herpes zoster neuralgia and post-herpetic neuralgia presented with higher concentrations [17]. In human immunodeficiency virus infection, galectin-3 has been shown to support the attachment of the virus to macrophages and CD4+ T cells [19,34]. Galectin-3 was induced in hepatitis B and C infections [35,36,37]. Human herpesvirus 1 infection increased the secretion of galectin-3 from cells [20]. Moreover, in herpesvirus infection, galectin-3 was involved in the regulation of adaptive immunity (it constrained the activation of CD8+ T-cells) [38]. The increased serum galectin-3 concentrations in severe SARS-CoV2 infection was consistent with the reports showing the induction or increased secretion of galectin-3 in response to infections. Of note, the serum concentrations of galectin-3 in the patients with moderate COVID-19 (without pneumonia and not requiring intensive care) did not differ as compared to healthy subjects. Increased serum galectin-3 in severe cases in correlation with increased concentrations of IL-6 and acute phase proteins was consistent with the cytokine storm observed in severe COVID-19 [39].

An interesting finding of the present study was the significant positive correlation between serum galectin-3 and the marker of endothelial dysfunction, sFlt-1. Although sFlt-1 is most studied as the laboratory marker of preeclampsia [40], we observed increased serum concentrations of sFlt-1 (together with angiopoietin-2) in acute systemic inflammation associated with severe acute pancreatitis [14]. Increased serum sFlt-1 may reflect the endothelial dysfunction induced by the hyperinflammation. This is in line with thrombotic complication and consumptive coagulopathy observed in severe COVID-19 [10]. Of interest, recent publications suggest that SARS-CoV-2 may also directly damage the endothelial cells, as they express ACE-2, and thus may be infected by the virus [8,9,10]. The damage of the cells expressing ACE-2 and the resulting ACE-2 deficiency lead to diminished anti-inflammatory signaling of angiotensin 1-7 [8,41]. In our patients with COVID-19, serum sFlt-1 was higher compared to controls, and the highest concentrations were associated with most severe disease course (i.e., the need for ICU admission). Although there are not many studies assessing the markers of endotheliopathy in COVID-19, the existing ones show the associations of such markers with disease severity [12,42,43]. However, to our knowledge, sFlt-1 has not been previously studied in COVID-19.

In our study, serum galectin-3, PTX-3, and sFlt-1 were interrelated. While increased CRP, produced by hepatocytes, reflects the systemic inflammatory response, the long pentraxin PTX-3 is instead produced locally in the sites of inflammation by the endothelial cells or macrophages [7]. In the experiments of Brunetta et al. [7], SARS-CoV-2 strongly induced PTX-3 expression in respiratory tract epithelial cells. Schirinzi et al. [6] observed increased PTX-3 early in the course of COVID-19 in patients admitted to the emergency department. The chronic endothelial dysfunction observed in patients with atherosclerosis and cardiovascular disease is associated with increased circulating PTX-3 [44,45,46]. Of note, atherosclerosis and cardiovascular disease are among the risk factors of severe COVID-19, and the chronic endothelial dysfunction predisposing to increased endothelial injury is suggested as the pathophysiologic link [8].

Serum galectin-3 has been proposed as an early marker of chronic inflammatory states associated with fibrosis, and of metabolic diseases and neoplasms [47,48]. It has been extensively studied as a marker of cardiac fibrosis, and in large cohorts, predicted incident heart failure and all-cause mortality [49,50]. The experimental data links galectin-3 to micro- and macrovascular complications of diabetes and to atherosclerosis [48,51]. In our study, serum galectin-3 was higher in overweight or obese patients and in those with diabetes. In addition, it was positively correlated with the Charlson comorbidity index. Still, serum galectin-3 predicted more severe COVID-19 independently of age and Charlson comorbidity index.

We found positive correlations between serum galectin-3 and the markers of tissue injury, including hemoglobin oxygen saturation, enzymes (LDH, GGT, aspartate aminotransferase), and urea. Galectin-3 negatively correlated with blood hemoglobin, hematocrit, and erythrocyte count. We may hypothesize that these correlations reflect the simultaneous association of galectin-3 and the mentioned markers with COVID-19 severity. However, we cannot exclude the possibility that galectin-3 is directly involved in tissue damage.

The main limitation of our preliminary study was the relatively small sample size and the fact that some laboratory tests were not performed in every patient (we used the data obtained during the first five days of routine hospital care). Nevertheless, our study showed that serum levels of galectin-3 were increased in severe COVID-19 in association with acute phase reactants, the marker of endothelial injury (sFlt-1), and the markers of tissue damage. This strengthens the hypothesis that galectin-3 may be involved in the pathomechanism of severe COVID-19. The emergence of multiple SARS-CoV-2 variants and the impact of vaccination both modify the course of COVID-19. Further studies are planned to confirm our preliminary findings and to verify the possible associations of galectin-3 with long-term consequences of COVID-19, including pulmonary fibrosis.

## Figures and Tables

**Table 1:** Demographic and clinical characteristics of the studied patients on admission. Quantitative data are shown as median (IQR) or mean (SD).

Characteristic	Values (n = 70)
Age, years	58 (49; 67)
Male sex, n (%)	27 (38.6)
BMI, kg/m^2^ *	28.4 (4.2)
Body temperature, °C	37.8 (36.4; 38.6)
Systolic blood pressure, mmHg	135 (20)
Diastolic blood pressure, mmHg	85 (12)
Heart rate, beats/min	89 (15)
Hemoglobin oxygen saturation, %	97 (94; 98)
Symptoms:	
Cough, n (%)	41 (58.6)
Weakness, n (%)	40 (57.1)
Dyspnea, n (%)	29 (41.4)
Muscle pain, n (%)	19 (27.1)
Headache or dizziness, n (%)	15 (21.4)
Loss of taste, n (%)	15 (21.4)
Loss of smell, n (%)	14 (20.0)
Sore throat, n (%)	13 (18.6)
Shivers, n (%)	12 (17.1)
Diarrhea, n (%)	12 (17.1)
Nausea or vomiting, n (%)	8 (11.4)
Abdominal pain, n (%)	4 (5.7)
Comorbidities:	
Hypertension, n (%)	44 (62.9)
Diabetes mellitus, n (%)	17 (24.3)
Thyroid disease, n (%)	15 (21.4)
COPD or asthma, n (%)	8 (11.4)
Charlson comorbidity index, points	2 (1; 4)

* BMI values were available in a subgroup of 40 patients. Abbreviations: BMI, body mass index; COPD, chronic obstructive pulmonary disease; n, number of patients.

**Table 2:** Laboratory results. Data are shown as median (IQR) or mean (SD).

Laboratory Test	n ^1^	Values	Reference Range
RBC, × 10^6^/µL	66	4.31 (4.07; 4.52)	F: 4.0–5.0; M: 4.5–5.9
Blood hemoglobin, g/dL	66	12.7 (12.3; 13.5)	F: 12–16; M: 14–18
Hematocrit, %	66	38.0 (36.4; 40.5)	F: 37–47; M: 40–54
WBC, × 10^3^/µL	66	5.96 (4.70; 6.69)	4.5–10.0
Neutrophils, × 10^3^/µL	62	3.49 (2.62; 4.11)	1.8–7.7
Lymphocytes, × 10^3^/µL	62	1.50 (1.25; 1.87)	1.0–4.5
Neutrophil/lymphocyte ratio	62	2.16 (1.55; 3.55)	Not available
Monocytes, × 10^3^/µL	62	0.51 (0.41; 0.64)	0.1–0.8
Platelet count, × 10^3^/µL	66	246 (202; 333)	140–440
Alanine aminotransferase, U/L	68	37 (20; 62)	F: 10–35; M: 10–50
Aspartate aminotransferase, U/L	66	26 (18; 47)	F: 10–35; M: 10–50
Gamma-glutamyl transpeptidase, U/L	64	40 (22; 80)	F: 5–36; M: 8–61
Alkaline phosphatase, U/L	65	64 (55; 88)	F: 35–104; M: 40–129
Lactate dehydrogenase, U/L	63	179 (130; 244)	F: 135–214; M: 135–225
Total bilirubin, µmol/L	65	6.40 (4.70; 8.56)	< 21.0
Glucose, mmol/L	61	5.37 (4.75; 6.38)	3.30–5.60
Urea, mmol/L	69	5.40 (4.24; 6.81)	2.76–8.07
Creatinine, µmol/L	68	67.6 (60.0; 88.8)	F: 44–80; M: 62–106
Lactate, mmol/L	6	1.20 (0.90; 1.50)	0.5–2.2
Albumin, g/L	63	38.8 (5.6)	35–52
Total protein, g/L	63	65.9 (6.1)	66–87
Sodium, mmol/L	63	140 (139; 142)	136–145
Potassium, mmol/L	68	4.53 (0.60)	3.50–5.10
Total cholesterol, mmol/L	62	4.78 (1.23)	3.2–5.2
HDL-cholesterol, mmol/L	62	1.04 (0.27)	F: > 1.2; M: > 1.0
LDL-cholesterol, mmol/L	62	2.61 (1.03)	< 3.4
Triglycerides, mmol/L	62	1.63 (1.33; 1.94)	< 2.26
Creatinine kinase, U/L	64	42 (29; 70)	F: 26–192; M: 39–308
Cardiac troponin I, ng/L	10	2.95 (2.00; 4.98)	< 47.3
Myoglobin, µg/L	7	46.2 (36.4; 103.8)	< 110.0
NT-proBNP, pg/mL	9	119 (80; 507)	< 125 (< 75 years); < 450 (≥ 75 years)
Iron, µmol/L	64	17.0 (11.6; 22.1)	5.83–34.5
Ferritin, µg/L	64	216 (123; 526)	13–400
Prothrombin time, s	24	10.9 (10.5; 11.4)	10.4–13.0
D-dimer, mg/L	37	0.48 (0.28; 1.15)	< 0.55
C-reactive protein, mg/L	69	4.79 (1.48; 23.90)	< 0.5
Procalcitonin, ng/mL	20	ND (ND; 0.08)	< 0.10
Interleukin-6, pg/mL	62	3.77 (1.50; 16.71)	< 7.0
Pentraxin-3, ng/mL	70	2.37 (2.02; 3.48)	1.47–4.25 ^2^
sFlt-1, pg/mL	64	128 (111; 148)	63.3–108.3 ^2,3^
Galectin-3, ng/mL	70	9.41 (7.74; 14.22)	5.49–12.77 ^2^

^1^ Number of patients with available test results; ^2^ range in a group of 10 (pentraxin-3 and galectin-3) or 21 (sFlt-1) healthy subjects; ^3^ statistically significant difference between patients and healthy subjects. Abbreviations: RBC, red blood cell count; WBC, white blood cell count; ND, non-detectable; HDL, high-density lipoprotein; LDL, low-density lipoprotein; NT-proBNP, N-terminal pro-B-type natriuretic peptide; sFlt-1, soluble fms-like tyrosine kinase-1; F, females; M, males.

**Table 3:** Statistically significant differences between patients who developed and did not develop COVID-19 pneumonia. Data are shown as median (IQR) or mean (SD).

Characteristic	Pneumonia (n = 23)	No Pneumonia (n = 47)	*p*
Body temperature, °C	38.5 (37.8; 39.0)	36.8 (36.3; 38.5)	0.007
Hemoglobin saturation, %	94 (88; 96)	97 (96; 98)	< 0.001
Platelet count, × 10^3^/µL	301 (223; 403)	232 (190; 300)	0.029
Gamma-glutamyl transpeptidase, U/L	66 (42; 198)	34 (21; 73)	0.035
Lactate dehydrogenase, U/L	221 (175; 316)	158 (124; 209)	0.005
Prothrombin time, s	11.4 (10.8; 12.1)	10.8 (10.5; 11.2)	0.047
Ferritin, µg/L	327 (197; 712)	176 (103; 351)	0.026
Interleukin-6, pg/mL	7.01 (2.54; 55.64)	2.86 (1.50; 10.45)	0.037
HDL-cholesterol, mmol/L	0.89 (0.78; 1.01)	1.04 (0.89; 1.26)	0.014
Total protein, g/L	63.3 (5.5)	67.1 (6.0)	0.031
Albumin, g/L	35.5 (5.9)	40.2 (4.9)	0.005
Galectin-3, ng/mL	13.30 (8.93; 17.38)	8.55 (6.73; 10.98)	0.001
Pentraxin-3, ng/mL	2.92 (2.19; 4.77)	2.28 (1.84; 3.21)	0.032

**Table 4:** Statistically significant differences between patients who required and did not require treatment in intensive care unit (ICU) during hospital stay. Data are shown as median (IQR) or mean (SD).

	ICU Treatment (n = 9)	No ICU Treatment (n = 61)	*p*
Diastolic blood pressure, mmHg	74 (8)	86 (12)	0.002
Hemoglobin saturation, %	92 (81; 95)	97 (95; 98)	0.003
Hematocrit, %	33.4 (29.5; 38.4)	38.2 (36.5; 40.7)	0.021
Hemoglobin, g/dL	11.3 (9.9; 12.6)	12.9 (12.3; 13.8)	0.014
RBC, × 10^6^/µL	3.66 (3.41; 4.27)	4.31 (4.08; 4.70)	0.007
WBC, × 10^3^/µL	10.60 (6.24; 11.46)	5.84 (4.45; 6.61)	0.002
Neutrophil count, × 10^3^/µL	5.73 (3.55; 8.53)	3.32 (2.56; 3.94)	0.044
Monocyte count, × 10^3^/µL	0.93 (0.81; 1.10)	0.50 (0.40; 0.58)	0.004
Platelet count, × 10^3^/µL	403 (272; 478)	232 (189; 308)	0.010
C-reactive protein, mg/L	18.2 (5.9; 71.1)	3.6 (1.2; 17.5)	0.009
Gamma-glutamyl transpeptidase, U/L	253 (117; 308)	35 (21; 74)	0.006
Lactate dehydrogenase, U/L	322 (225; 548)	164 (129; 221)	0.001
D-dimer, mg/L	1.62 (0.90; 2.14)	0.32 (0.25; 0.78)	0.003
Ferritin, µg/L	766 (526; 1235)	209 (111; 474)	0.007
Interleukin-6, pg/mL	16.98 (6.46; 99.90)	3.42 (1.50; 15.92)	0.006
HDL-cholesterol, mmol/L	0.82 (0.69; 0.90)	1.03 (0.88; 1.20)	0.009
Albumin, g/L	32 (6)	40 (5)	0.006
sFlt-1, pg/mL	183 (131; 260)	124 (107; 143)	0.006
Galectin-3, ng/mL	23.46 (15.51; 27.80)	8.93 (7.58; 12.97)	0.001
Pentraxin-3, ng/mL	4.77 (2.92; 8.17)	2.30 (1.88; 3.21)	0.001

**Table 5:** Statistically significant correlations between galectin-3 and the selected variables. We report the Spearman rank correlation coefficients (R) and *p*-values.

Variable	Galectin-3	
	R	*p*
Diastolic blood pressure	−0.30	0.012
Hemoglobin saturation	−0.36	0.002
BMI	0.43	0.006
Charlson comorbidity index	0.27	0.021
Hematocrit	−0.33	0.007
Hemoglobin	−0.28	0.022
RBC	−0.42	< 0.001
Aspartate aminotransferase	0.25	0.040
Gamma-glutamyl transpeptidase	0.26	0.036
Lactate dehydrogenase	0.55	< 0.001
Sodium	−0.25	0.049
Urea	0.33	0.006
HDL-cholesterol	−0.38	0.003
Triglycerides	0.27	0.031
Total protein	−0.29	0.022
Albumin	−0.41	0.001
Ferritin	0.51	< 0.001
C-reactive protein	0.28	0.018
Interleukin-6	0.57	< 0.001
Pentraxin-3	0.54	< 0.001
sFlt-1	0.48	< 0.001

**Table 6:** Multiple logistic regression models showing the associations between galectin-3 and COVID-19 pneumonia and the need for intensive care. Odds ratios (OR) with 95% confidence intervals (95% CI) are reported per 1 unit increase in the value of each independent variable.

Independent Variable	Dependent Variable			
	Pneumonia		Treatment in ICU	
	OR (95% CI)	*p*	OR (95% CI)	*p*
Galectin-3	1.19 (1.06–1.34)	0.002	1.31 (1.11–1.53)	0.001
Age	1.01 (0.95–1.07)	0.7	1.00 (0.91–1.11)	0.9
Charlson comorbidity index	0.86 (0.59–1.24)	0.4	0.94 (0.54–1.65)	0.8

Abbreviations: OR, odds ratio; CI, confidence interval.

## Data Availability

Data supporting the results are available from the corresponding author upon reasonable request.
